# Social orienting of children with autism to facial expressions and speech: a study with a wearable eye-tracker in naturalistic settings

**DOI:** 10.3389/fpsyg.2013.00840

**Published:** 2013-11-20

**Authors:** Silvia Magrelli, Patrick Jermann, Basilio Noris, François Ansermet, François Hentsch, Jacqueline Nadel, Aude Billard

**Affiliations:** ^1^Learning Algorithms and Systems Laboratory, École Polytechnique Fédérale de LausanneLausanne, Switzerland; ^2^Center for Digital Education, École Polytechnique Fédérale de LausanneLausanne, Switzerland; ^3^Service of Child and Adolescent Psychiatry, Department of Child and Adolescent Medicine, Hôpitaux Universitaires de GenèveGenève, Switzerland; ^4^Emotion Center, CNRS and The Universite Pierre et Marie CuriePitié-Salpêtrière, Paris, France.

**Keywords:** autism spectrum conditions, eye-tracking, social orienting, overt attention, facial expressions of emotion, speech

## Abstract

This study investigates attention orienting to social stimuli in children with Autism Spectrum Conditions (ASC) during dyadic social interactions taking place in real-life settings. We study the effect of social cues that differ in complexity and distinguish between social cues produced by facial expressions of emotion and those produced during speech. We record the children's gazes using a head-mounted eye-tracking device and report on a detailed and quantitative analysis of the motion of the gaze in response to the social cues. The study encompasses a group of children with ASC from 2 to 11-years old (*n* = 14) and a group of typically developing (TD) children (*n* = 17) between 3 and 6-years old. While the two groups orient overtly to facial expressions, children with ASC do so to a lesser extent. Children with ASC differ importantly from TD children in the way they respond to speech cues, displaying little overt shifting of attention to speaking faces. When children with ASC orient to facial expressions, they show reaction times and first fixation lengths similar to those presented by TD children. However, children with ASC orient to speaking faces slower than TD children. These results support the hypothesis that individuals affected by ASC have difficulties processing complex social sounds and detecting intermodal correspondence between facial and vocal information. It also corroborates evidence that people with ASC show reduced overt attention toward social stimuli.

## 1. Introduction

Autism Spectrum Conditions (ASC, Baron-Cohen et al., 2009) encompass a set of neuro-developmental disorders that are typically manifested in childhood. These are characterized by qualitative impairments in social communication and interaction and by the presence of restricted and repetitive behaviors (APA, 2000). Research, in the last decades, in the attempt to define the nature of the development of social communication abilities in autism, identified the key role played by social attention impairments (Dawson et al., 2012). Deficits of social attention in ASC include difficulties with orienting (i.e., shifting the attention) in response to social stimuli, especially to spoken language (O'Connor, 2012). In the literature on autism, both overt and covert orienting have been intensively studied. Whereas, overt attention orienting is visible to external observers through eye movements and head turns, covert attention orienting consists of a shift of spatial attention that cannot be directly perceived.

Lack of overt social orienting is considered among the most salient and specific features able to distinguish infants later diagnosed with ASC from other infants at high risk of developmental impairments or delay (Swettenham et al., 1998; Zwaigenbaum et al., 2005; Rogers, 2009; Zwaigenbaum et al., 2009).

Retrospective analyses of home videotapes or parental descriptions of children's behavior within the first 2 years of life prior to diagnosis (Wimpory et al., 2000; Werner and Dawson, 2005) were particularly significant in the discovery of peculiarities in social attention in infants with autism, because they provide a detailed account of the behavior of these children in real-life settings where manifestations of social impairments are more evident (Simmons et al., 2009). Specifically, studies based on the analysis of home videotapes provided early evidence that infants with ASC are less likely to overtly orient to their name being called at 8–10 months (Werner et al., 2000), 9–12 months (Baranek, 1999) and 1 year of age (Osterling and Dawson, 1994; Osterling et al., 2002) relative to both age-matched typically developing (TD) controls and infants with mental retardation (Osterling et al., 2002). Similar results have been obtained in 3–4 year old (Dawson et al., 2004) and 5–6 year old children with ASC (Dawson et al., 1998).

Studies in visual orienting of attention in autism that have been conducted in laboratory settings used mostly cueing tasks based on the Posner's test (Posner, 1980). The Posner's test is a spatial-cueing paradigm, originally proposed by Posner to address covert attention orienting but that can be also used to assess overt attention orienting. In the Posner's test, subjects are exposed to either valid or invalid cues (e.g., arrows, flashlights, eye-gaze) prior to perform a visual selection task. In these experiments, the cue appears on a monitor typically 100 milliseconds (ms) before the target and, if the cue is valid, it appears at the same spatial location as the target. When presented with a valid cue, subjects tend to attend the target with faster reaction times and more accurately than if the cue was invalid, i.e., had appeared in another location (e.g., Müller and Humphreys, 1991). This can be due to the fact that attention is attracted reflexively to the spatial location of the directed cue and remains covertly toward that position even though an intermediate repositioning of the gaze in the center of the screen is required by the task (Posner, 1980; Friesen and Kingstone, 1998; Driver et al., 1999; Langton and Bruce, 1999). Therefore, the analysis of the reaction times to looking at the target, provides information on whether the subjects covertly oriented the focus of their attention to the expected location suggested by the cue.

Studies on social visual orienting based on the Posner's cueing paradigm adopted a social spatial cue such as a person with her eyes gazing to a specific location (Greene et al., 2011). In artificial settings the evidence of impaired social orienting is mixed. The majority of studies conducted with a Posner's like paradigm reported intact overt orienting in the direction of the perceived gaze in ASC with both dyanamic and static gaze cues (Chawarska et al., 2003; Okada et al., 2003; Swettenham et al., 2003; Kylliäinen and Hietanen, 2004; DeJong et al., 2008; Rutherford and Krysko, 2008; Uono et al., 2009; Kuhn et al., 2010). Few studies observed impaired gaze-cueing in autism (Johnson et al., 2005; Ristic et al., 2005; Goldberg et al., 2008).

As suggested by Birmingham et al. (2012), experimental designs that tightly control the cueing paradigms may not capture fundamental mechanisms underlying attention orienting in real life situations. According to the authors, as subjects are instructed by the experimenter and the social setting is completely controlled, the opportunity for spontaneously orienting to social cues and the ambiguity and complexity involved in natural situations are completely removed. This would explain the contrast between the evidence of intact social orienting in autism reported by artificial cueing paradigms and the findings that report difficulties in social attention in more naturalistic social environments (Nadel and Butterworth, 1999; Klin et al., 2002).

The neuro-functional impairments underlying the behavioral patterns at the basis of the peculiarities in social orienting of individuals with ASC, have yet to be understood. Several hypotheses have been raised, with one possible direction of investigation residing in the study of the sensory processing modules that provide input information to the attention processes. In fact, several studies have reported the presence of abnormal processing of both the auditory and visual social stimuli (Baron-Cohen, 1989; Dawson et al., 1998; Baron-Cohen et al., 2001; Rutherford et al., 2002; Dawson et al., 2004, 2005).

Findings show that individuals with ASC are most likely to exhibit impairments in perception of complex auditory information and that these deficits are more pronounced for speech than for non-speech stimuli (Klin, 1991; Ĉeponienė et al., 2003; Kuhl et al., 2005; Kujala et al., 2005; Oram-Cardy et al., 2005; Whitehouse and Bishop, 2008; see Peppe and McCann, 2003, for a review).

Research has also separately investigated the nature of impairments of emotional identification and perception through facial emotion recognition tasks. Though anomalies in scanning patterns of faces have been demonstrated in autism, there are mixed findings on the ability to both process faces (see Simmons et al., 2009 for a review) and recognize emotions from facial expressions (see Harms et al., 2010 for a review). In their review, Harms et al. (2010) hypothesized that the discrepancies in findings depend on the task demands, social stimuli types and the demographic factors of the participants.

In the present work, we hence set forth to study social orienting in children with ASC when engaged in naturalistic playing interactions with an adult. To investigate the presence of shifting of attention to human faces, we conducted a systematic analysis of the temporal gaze patterns directed to the adult's face before and after the onset of the presented social stimuli. The onset of the stimuli was used as a social cue for the presented social stimuli. We used a paradigm that is completely unconstrained and natural. This was previously adopted by studies that investigated social orienting in autism conducted by a retrospective analysis of home videotapes (Osterling and Dawson, 1994; Dawson et al., 1998; Baranek, 1999; Werner et al., 2000; Wimpory et al., 2000; Osterling et al., 2002; Dawson et al., 2004; Werner and Dawson, 2005). Moreover, we conducted a differentiated analysis in two social conditions, namely when the adult was addressing the child either verbally or through facial expressions of emotion.

Facial expressions of emotion involve solely the processing of visual information (the emotion of the eye, the position of the mouth, etc.) therefore, they are mono-modal social cues. Speech, on the other hand, requires the use of two modes (Massaro and Bosseler, 2003). The combination of both a visual stimulus (e.g., movements of the mouth, the intensity of the gaze while the person is speaking, facial expressions) and an auditory stimulus (the sound and the tone of the speech, etc) are conveyed to the interlocutor.

Given the ample evidence that social impairments in ASC are aggravated by the complexity of the social stimuli and because both children and adults affected by ASC present impairments in detecting intermodal correspondence between facial and vocal information (de Gelder et al., 1991; Loveland et al., 1995; Mongillo et al., 2008), we hypothesized that children with ASC would present a differentiated response to the two types of events considered here. Specifically, we expected that in children with ASC, the response to an adult's use of speech and facial expressions to convey emotions will be less intense than to facial expressions alone.

We used the WearCam (Piccardi et al., 2007; Noris et al., 2011), a light-weight and unobtrusive eye-tracking technology specifically designed to span a broad region of the child's visual field. The WearCam technology enables us to directly quantify the extent to which a subject spontaneously maintains a target in a specific location of the visual field of view thanks to the broad region that is recorded. Our system adopts the child's point of view while enabling the gaze patterns to be independently analyzed from the direction of her head.

## 2. Materials and methods

### 2.1. Participants

A group of children with ASC from 2 to 11-years old (*n* = 14) and a group of typically developing (TD) children (*n* = 17) between 3 and 6-years old took part in the experiment (see Table 1).

**Table 1:** **Participants information**.

**ASC**	**CARS**	**Gender**	**Chron. Age**		**Chron. Age**	**Gender**	**TD**
1	34.5	M	1.83		3.08	F	1
2	34.5	M	4.08		3.67	F	2
3	44	M	4.17		4.42	F	3
4	35.5	F	4.25		4.42	M	4
5	34	M	4.67		4.75	F	5
6	46	M	5.92		5.58	F	6
7	47	F	6.58		5.92	F	7
8	44	M	4.08		6.08	F	8
9	36.5	F	8		2.91	M	9
10	49	M	7.67		2.75	M	10
11	47	F	10.58		3.0	M	11
12	33	F	7.17		3.16	M	12
13	30	M	10.0		2.33	M	13
14	35.5	M	5.83		2.83	M	14
					5.08	M	15
					2.41	M	16
					5.08	F	17
	39.32		6.06	Mean	3.99		
	6.44		2.46	Std	1.27		

Participants in the ASC group were recruited at the Geneva University Hospital and through the Autisme Suisse Romande Association. The diagnosis of autism was established using the Autism Diagnostic Interview-Revised (ADI-R) (Lord et al., 1994), administrated by expert clinicians. In addition, the children in the ASC group were scored with the CARS (Schopler et al., 1980) as presenting mild to severe autism (*M* = 39.58, *SD* = 6.66, range: 30–49). Their mean chronological age was 6.08 years (*SD* = 2.03, range: 3.08–6.08). The control participants were volunteers recruited at the day-care facilities at EPFL. The mean chronological age of the TD children was 3.99 years (*SD* = 1.27, range: 2.33–6.1), see Table 1. All parents provided written informed consent including the permission to use the video recordings for scientific publications. The experimental procedures and form of consent were approved by the ethics committee of the University Hospital of Geneva.

If a participant was uncooperative during the first visit, we scheduled a second visit within 2 weeks after the first one. Four children in the recruited ASC group were uncooperative during the first visit, but they subsequently cooperated during the second visit and were included in the study. We did not include in the study an infant with autism who was uncooperative in both the visits, therefore another child with ASC was recruited to complete the group. None of the subjects failed to complete the experiment due to the failure in the calibration or other technical problems. None of the control subjects refused to wear the eye-tracking device.

### 2.2. Apparatus: the WearCam system

We recorded the experimental sessions using the WearCam, a wearable eye-tracking system developed at EPFL and specifically designed for children (Piccardi et al., 2007; Noris et al., 2011).

**Figure 1:**
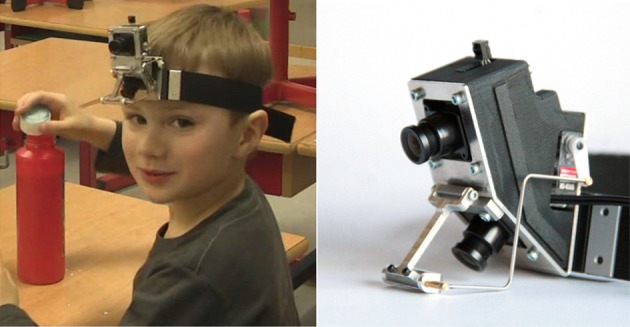
**The WearCam consists of a wearable elastic strap (right) mounted with a pair of cameras**. The top camera gives a view of the “interaction” zone while the bottom camera gives a view of the “lower zone.” A mirror mounted on the bottom camera reflects the child's eyes for tracking the user's gaze (right).

**Figure 2:**
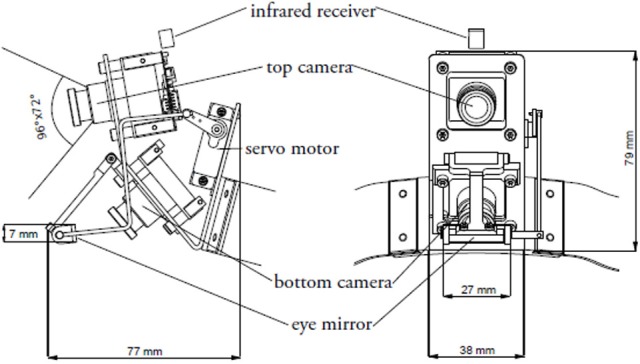
**Schematics of the WearCam: the two CCD cameras, servo motor and mirror for reflecting the child's eyes**.

The WearCam (see Figures 1, 2) is an head-mounted device comprised of two Sony Super HAD CCD cameras, mounted one on top of the other, and a mirror driven by a servo motor. Adjustable straps allow to secure the device either on the child's head or on a cap. The device is lightweight and unobtrusive, weighing only 180 gr and therefore particularly suited for children.

The first camera is pointed forward and aligned with the head of the wearer in order to capture the region of the visible field of view when the eyes are aligned with the forehead. Thus, this camera allows the monitoring of what is happening in the environment and specifically to record the interaction between the child and the adult.

The second camera points slightly downwards, capturing the lower part of their field of view (e.g., the region where the child focuses her attention while manipulating objects with her hands). The bottom camera also captures the image of the mirror that reflects the child's eyes. The image of the child's eyes is used during *post-hoc* analysis of the video to track the child's gaze. The servo motor is remotely controlled in order to align the mirror with the eyes of the wearer and so as to prevent the manual adjustment of the cameras' position.

The two cameras are angled at 30° and therefore they record overlapping images. In this way, a video image from the upper camera can provide the region of the child's field of view that is occluded by the mirror in the image from the lower camera. Therefore, the system allows to extract synchronously the image of the child's eyes and the scene the child is looking at in the corresponding field of view.

Each camera captures a visual image covering a range of 96° by 72°. However, after removing the mirror from the top camera's image the whole image available to the system covers an angle of 96° by 96°. The WearCam records 384 × 576 MJPEG video images at a 25-Hz-frame rate. Therefore, image data are sampled every 40 ms. The accuracy of the WearCam eye-tracker was assessed in a previous study by Noris et al. (2011), to obtain an angular precision of 2.42° with TD children and of 1.60° with TD adults.

The calibration with the WearCam can be performed both on site (Noris et al., 2008) and off-line (Noris et al., 2011). During the present study, we adopted the off-line calibration procedure since it does not require the child to actively participate. In this way, the experiments could be started as soon as the elastic strap was secured to the child's head and the mirror was correctly oriented for the child's eyes to be visible in the camera's image. The child is facilitated to focus her attention solely on the playing session with the adult: the mirror is the only part of the eye-tracker she is aware of and calibration is performed off-line. This prevented us from having to stop the experiment when the Wearcam slightly moved during the study. Two trained raters, unaware of the purpose of the study and of the diagnosis, watched each video sequence with a custom-made software and collected from 70 to 100 calibration points following the procedure described in the study of Noris et al. (2011). The whole process lasted 10–15 min per subject.

The WearCam eye-tracker uses features based on the appearance of the eyes, such as the shape and shading of the eyelids, to map the coordinates of the gaze in the field of view. Mapping the calibration points to the complete image of the visual field returned by the cameras is done through non-linear regression using support vector regression (SVR) (Schölkopf and Smola, 2002). SVR is trained separately on the horizontal and vertical dimensions of the image. Then, a non-linear mapping between the 2-dimensional coordinates on the image of the field of view and on the image of the eyes, recorded by the WearCam, is computed. The WearCam enables to track the eyes even when geometrical elements are occluded (e.g., when the child is looking downwards and the iris is not completely visible). This is due to the fact that the SVR mapping exploits both geometrical elements, such as position of the iris and pupil, and non-geometrical features, such as the shape of eyelashes and shading on the eyelids. To adapt the SVR model to the fact that the eyes appeared differently in the image when the Wearcam slightly moves, we gather a few additional calibration points using the part of the video recordings that follows the possible displacement of the elastic strap secured on the head.

### 2.3. Procedure

Each child participated in a recording session that lasted a maximum of 20 min. During the experiments, the WearCam apparatus was mounted in a child's hat and secured via an elastic strap. The camera recorded the dyadic play interactions between the child and an adult in places familiar to the subjects.

The experimental task consisted of two play sessions of 10 min that differed for the presence of different taking turn games: blowing soap bubbles and making Play-Doh. The person that interacted with the children was a carer, who had become familiar to the children thanks to the numerous visits done to the parent's house prior to the study. This person will be referred to as the adult in the rest of the manuscript.

Experiments took place with only the child and the adult present, while monitored by a technician, hidden in an adjacent room. Therefore, children only had the opportunity to look at the face of the adult.

The adult was instructed to make sure that they took turns blowing bubbles and playing with Play-Doh. During each session, the adult interacted with the child while sitting at a table facing the child. This guaranteed that the distance (~80 cm) between the adult and the children was preserved all along the experiment.

### 2.4. Data processing

We designed a simple behavioral coding system for studying the gaze behaviors adopted by the children during the naturalistic interactions and for examining whether they were affected by the complexity of the social stimuli.

Double blinded raters, blind to the diagnosis and to the aim of the study, were asked to label the video sequences in which the adult interacting with the child was either speaking or making facial expressions of surprise or smile, see Table 2. Both the social cues where labeled only when the adults showed the explicit intent of communicating with the child. Therefore, for an event to be labeled, respectively, as speech or a facial expression, the adult had to look directly in the child's eyes and, either speak or show a feeling of surprise or happiness. Video sequences in which the adult was making neutral faces were also coded.

**Table 2:** **Inter-rater reliability**.

**Faces**	**Speech**	**Facial expressions**	**Objects**
0.91	0.95	0.88	0.97

Inter-rater correlation for the output of the labeling process.

**Figure 3:**
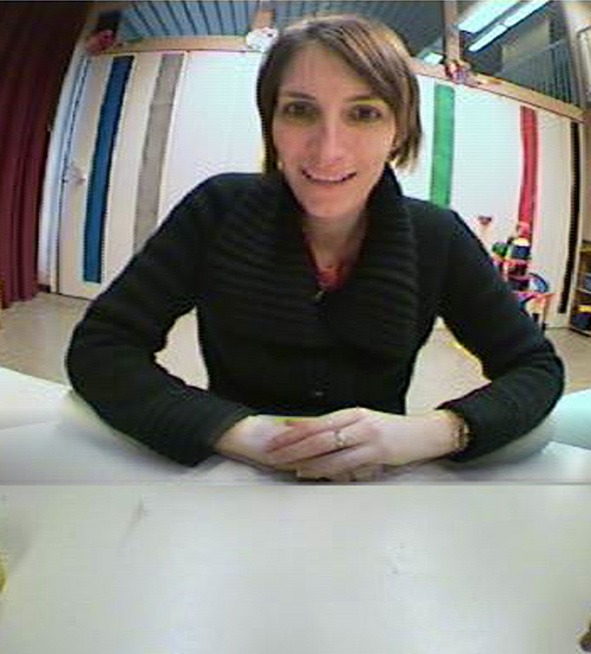
**Image of the interaction with the experimenter recorded from the WearCam**. The shown image was taken into account in the statistical analysis. In fact, the adult interacting with the child makes a facial expression (smile) that is directed to the child wearing the eye-tracker. Moreover, there is no object of interaction visible in the child's field of view.

Moreover, we used a dedicated software (Noris et al., 2007) to perform automatic tracking of areas of interest (AOIs). The AOIs consisted of the regions that included the adult's face or the object of interaction. Each region was delineated with a bounding box, see Figure 3. The automatic tracker has an accuracy of about 80%. To compensate for this, the human raters reviewed the output of the video tracking system and added entries where the detection was incorrect. Each subject's data were coded at a time by the two raters.

After processing the data, we isolated and analyzed two different sets of video sequences that correspond to two different conditions:

1. The facial expression condition: sequence of video frames in which the child held the adult's face in the field of view for a period of 1200 ms before and after the onset of the adult's facial expressions; and for which no toy object was present in the field of view. The sequence of video frames before the onset of the facial expression displays the adult's face with a neutral facial expression. The sequence of video frames after the onset of the facial expression displays the adult's face with a facial expression (i.e., smile or surprise).
2. The speaking condition: the sequence of video frames in which the child held the adult's face in the field of view for a period of 1200 ms before and after the onset of the adult's speech; and during which no toy was present in the field of view. The sequence of video frames before the onset of the speech displays the adult's face with a neutral facial expression. The sequence of video frames after the onset of the speech displays the adult's face speaking. We herein clarify that the speech also involves expressing emotions through facial expressions; therefore, in this condition is evaluated the response to speech and to the facial expressions that are naturally produced during speech.

The number of segments is balanced between the two groups, in both conditions (facial expressions condition: TD: *M* = 2.14%, *SD* = 2.00; ASD: *M* = 3.37%, *SD* = 5.43, *df* = 132, *p* = 0.4584, AIC = 1350.72; BIC = 1363.67; logLik = −671.36; speaking condition: TD: *M* = 3.25%, *SD* = 3.19; ASD: *M* = 3.59%, *SD* = 3.21, *df* = 132, *p* =.297, AIC = 734.33; BIC = 745.86; logLik = −363.17).

We took care that video segments considered in the analysis did not temporally overlap. Specifically, each video segment is both preceded and followed by an interval of at least 200 ms in which the adult's face is either neutral or not present in the video scene. In this way we ensure that all the segments were distinct events and would not induce cross-effect.

The selected video sequences encompassed solely scenes where none of the experimental toys were visible in the field of view of the camera. The rationale behind this selection is to avoid bias on the measure of the gaze: children could have been focusing on objects present in their field of view rather than faces. By avoiding distractors, we can precisely describe behaviors in response to social cues.

The neutral faces before the onset of the social cues in both conditions are a useful term of comparison for the gaze patterns after the onset of the social cue.

Note that, in this study, we did not distinguish between different facial expressions. Although it could be interesting to analyze whether gaze patterns could be classified according to the emotions behind facial expressions, we were able to collect only facial expressions of happiness, namely: smiles and facial expressions of surprise. Indeed, during the experiments, the adults displayed only facial expressions with positive rewards, as would be naturally expected from a play session. An in-depth analysis of response on attention behavior to different facial expressions or speech acts would require building a specific protocol that balances the frequencies of events such as the facial expressions across the experiments. This would constrain the interactions between the children and the adults, thus reducing the spontaneity of the responses to socially demanding situations in which we are interested.

We have imposed a threshold of 1200 ms to the durations of the segments to be analyzed in the facial expression condition, given the third quartile of the distribution of these events (*M* = 730.8 ms, *Q3* = 1208.8 ms). The same threshold has been applied also to the speaking condition in order to guarantee compatibility in the tests. This leads to a total of 60 frames divided equally between the period prior and posterior to the onset of the social cues.

This duration was based on evidence from the literature that response to facial expression of emotion would take place within 300–700 ms on average. Schmidt et al. (2003) study the duration and the temporal patterning of facial movements in the onset of spontaneous facial expressions in two social contexts.

In their study Schmidt et al. (2003) find that viewers tend to respond to facial expressions by activating their own facial muscles as early as 300–400 ms after viewing a smile. Therefore, the authors suggest that the reaction time to facial expressions is most likely to be rapid and to appear in response to the onset of expressions, rather than other phases of the display. In fact, the smile onset presents the most prominent change in appearance of the face as perceived by human observers (Leonard et al., 1991). Whereas, smile onsets last at least 700 ms on average (Bugental, 1986), spontaneous smiles typically last for at least an average of 3000–4000 ms (Frank et al., 1993).

We first tracked and recorded the eye movements in the image assembled by the eye-tracking framework of the WearCam. Then, we computed the distance of the locus of the eye position from the Area of Interest (AOI) of the adult's face, tracked during the labeling process. Specifically, the distance of the gaze to face is computed as the distance in visual eccentricities from the point of the gaze to 2/3rds of the width of the face AOI.

In order to study visual orienting, we shaped the analysis on temporal trend of the distance between the gaze of children and the adult's face right before and after the trigger of social cues (facial expressions and speech).

For each participant, and separately for both experimental conditions, we collected the durations of the first fixations (FFLs) to faces and the reaction times (RTs). RT consists in the time interval between the onset of the facial expression and the beginning of the first fixation. We excluded from the analysis fixations that occurred too late, i.e., RT that exceeded 1200 ms and could no longer be related explicitly to the occurrence of the social cue. Moreover, we did not take into account anticipatory fixations (i.e., when children fixated on the adult's face just before the onset of the social cue.

### 2.5. Data analysis

In order to characterize the attention behaviors of children with respect to social events, we considered the segments containing the neutral expressions and the onset of the presented social events as positive cues for the targets: the adults' faces during the intervals in which the adults were either addressing the children verbally or making facial expressions while no distractors were present in the child's field of view. One marker of overt attention orienting in our study would, hence, be an episode when the child shifts his gaze toward the adult's face, right after the adult started speaking or making a facial expression. To look out for such markers, we hence computed the gaze-to-face distance over time, just prior and after the onset of the social cue. In this way, we can detect whether the group of children with ASC tend to shift their attention to the adult's face in response to social cues. We specifically retained only the video segments that contain the social cues (facial expressions and speech) and that follow immediately after a segment in which the adult's face was neutral.

Note that, in our experiments, the children were not explicitly asked to look at the adult's face, as opposed to experiments conducted in laboratory settings, as we wished to study spontaneous attention shifts in children in naturalistic interactions.

Similarly to the body of works in overt and covert attention orienting in both TD people (see Carrasco (2011) for a review on visual attention) and in individuals affected by ASC (Chawarska et al., 2003; Swettenham et al., 2003; Iarocci and Burack, 2004; Senju et al., 2004; Ristic et al., 2005; Vlamings et al., 2005), we analyze the reaction times to orient to the target in order to investigate possible differences between children with ASC and TD children.

#### 2.5.1. Assessment of attention shifts

**Figure 4:**
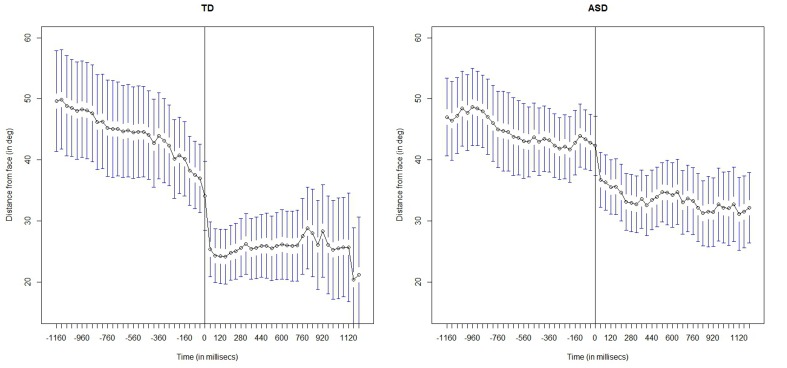
**Facial expression condition: frame-by-frame plot of the face-to-gaze distance, for the TD children (on the Left) and for the children with ASC (on the Right)**. Mean and quartiles of the temporal profiles the viewing patterns are depicted for each frame, 1200 s (30 frames) before and after the adult's onset of the facial expression. The vertical line in the middle highlight the onset of the social cue

First, we report and describe the pattern followed by the gaze for 1200 ms prior to and after the onset of the two social stimuli, i.e., facial expressions and speaking. The Figures 4, 6 show the mean distance gaze-to-face for both children with ASC and TD children. The herein presented analysis has been conducted using the R language for statistical computing. Mixed-effect model regression using the maximum likelihood method (ML) was executed by using the *lme* package in R.

A series of mixed-effect linear models were evaluated in order to assess the effect of the onset of the social cue (onset factor, ONS), autism (autism factor, AUT), chronological age (chronological age factor, AGE), task (task factor, TASK) and gender (GEN) on the temporal trend of the gaze-to-face distance after the social cues. Mixed-effect linear models were also analyzed for assessing the effect of autism (autism factor, AUT), chronological age (chronological age factor, AGE), task (task factor, TASK) and gender (GEN) on reaction times (RTs) and first fixations lengths (FFLs). The subject ID of the children was used as a random factor to account for the dependence between measures taken from the same individual. The models are nested and differ only for the random effects.

Nested models of increasing complexity are compared in order to identify the best one fitting the data. For this operation we employed forward stepwise model selection based on Akaike Information Criterion (AIC), the Bayesian Information Criterion (BIC), the log-likelihood (LogLik) and the log-likelihood ratio test (L. Ratio test) criteria with the command ANOVA(m0, m1, m2) in R. For the sake of conciseness we mainly report on the results of the comparison of the statistical significant models.

The AIC, the BIC, the logLik and the L. Ratio test are reported. The AIC value corrects the log-likelihood statistic for the number of estimated parameters. The BIC value corrects for the number of observations and the number of estimated parameters.

The information criteria together with log-likelihood statistics provide a way to assess the fit of a model based on its optimum log-likelihood value. However, as suggested by (Gurka, 2006) no one information criterion stands apart as the best criterion to be used in the selection Linear Mixed Models (LMMs). The L. Ratio test is used to compare the fit of two models, whereas one is nested within the other one.

## 3. Results

### 3.1. Spatio-temporal characteristics of gaze patterns in response to facial expressions

Figure 4 shows the mean distance gaze-to-face computed frame by frame for both children with ASC and TD children; before the onset, i.e., when the adult's face is neutral and after the onset, i.e., when the adult makes a facial expression.

By visual inspection of the graph, we immediately observe that after the onset of facial expression, both groups start to look more at the adult's face than before the onset of the social cue. This means that the children in both groups overtly shift their attention to the adult's face. However, there is a marked difference between the two groups: the children with ASC seem to look less at faces than TD children. In fact, after the onset, the mean gaze-to-face distance in children with autism looks greater than that of the TD children.

We conducted a linear mixed-effect regression analysis to verify these observations quantitatively and we report results below. Precisely, we computed liner mixed-effect models to assess the effect of the onset of the facial expression (onset factor, ONS), autism (autism factor, AUT), chronological age (chronological age factor, AGE), and task (task factor, TASK), see Table 3. The onset factor (ONS) separates all gaze-to-face distances into two groups: before the onset and after the onset. The subject ID of the children was used as a random factor to account for the dependence between measures taken from the same individual.

**Table 3:** **Facial expression condition: best fit mixed-effect models**.

	**Model**	***df***	**AIC**	**BIC**	**logLik**	**Test**	**L.Ratio**	***p*-value**
m1	Null Model	3	84641.50	84662.85	−42317.75			
m2	ONS	4	83937.46	83965.91	−41964.73	m1 vs. m2	706.04	<0.0001
m3	ONS + AUT	5	83934.97	83970.54	−41962.49	m2 vs. m3	4.49	0.034
m4	ONS ^*^ AUT	6	83894.47	83937.16	−41941.24	m3 vs. m4	42.5	<0.0001
m5	ONS ^*^ AUT + TASK	8	83854.35	83911.26	−41919.17	m4 vs. m5	47.83	<0.0001
m6	ONS ^*^ AUT ^*^ TASK	14	83661.41	83761.01	−41816.70	m5 vs. m6	204.94	<0.0001

Mean distance before and after the onset. Mixed-effect models providing the best fit for explaining the face-to-gaze distance levels, in the facial expression condition. The analysis is performed by taking into account the mean distance before and after the onset of the facial expression. Fixed effects: ONS, onset of the facial expression; AUT, autism; TASK, game (Play-Doh or bubbles). The model provided by ONS best improves the quality of the fit compared to the null model. Therefore, most of the differences in the gaze-to-face distances depend on whether they occurred before or after the onsets of the facial expressions.

We observe that the onset factor (ONS) significantly improves the quality of the fit compared to the null model, according to the BIC, AIC (i.e., lower is better) and log likelihood criteria (i.e., higher is better).

Thus, the best explanation for the differences in the gaze-to-face distances, is accounted for by the onset factor. Specifically, the set of gaze-to-face distances taken before the onset significantly differs (*p* < 0.0001) from the set of measures taken after the onset.

The autism factor (AUT) slightly increases the goodness of the model. Therefore, part of the difference (*p* = 0.0342) between the temporal trends is also explained by the diagnosis. This confirms the observation that the children with ASC look less at faces than TD children.

In addition, we report a significant interaction between onset and autism (ONS^*^AUT). By considering both the interactions between the onset and autism (ONS^*^AUT) and between autism and task (AUT^*^TASK), we obtain a significant improvement in the quality of the fit with respect to the nested models. This indicates that the gaze-to-face distance is affected by the autism condition and the type of task, in both the periods prior and following the onset of the facial expression.

To analyze the gaze-to-face distance, we further employed a linear mixed-effect regression, frame by frame, after the onset, separately. The analysis frame by frame considers the set of measures on the face-to-gaze distance in each specific frame, i.e., for each interval of 40 ms (the sampling period of the cameras). The model embeds two predictors. The autism categorical factor (AUT), combined with the temporal distance from the onset of the facial expression (frameID). Given the nature of the test, the user identifier was accounted for as a grouping factor. The results are shown in Tables 4, 5 for the interval immediately after the onset of the facial expression.

**Table 4:** **Facial expression condition**.

**After the onset**								
	**Model**	***df***	**AIC**	**BIC**	**logLik**	**Test**	**L.Ratio**	***p*-value**
m1	Null Model	3	40817.84	40837.12	−20405.92			
m2	AUT	4	40810.12	40835.84	−20401.06	m1 vs. m2	9.71	0.0018
m3	AUT + frameID	5	40811.53	40843.68	−20400.76	m2 vs. m3	0.59	0.4410
m4	AUT ^*^ frameID	6	40810.54	40849.11	−20399.27	m3 vs. m4	2.99	0.0836

Mixed-effect models. Frame by frame analysis. Mixed-effect models for the distance face-to-gaze level, in the facial expression condition, before and after the onset of the social cue. The analysis is performed frame by frame, (a frame corresponds to 40 ms). Fixed effects: frameID, temporal distance from the onset of the facial expression, expressed in frame number; AUT, autism. The model provided by AUT is the best explanation for the differences in gaze-to-face distances present in the data, after the onset.

**Table 5:** **Facial expression condition**.

**After the Onset**					
	**Value**	**Std.Error**	***df***	***t*-value**	***p*-value**
(Intercept)	22.00	3.37	4553	6.53	<0.0001
AUT	16.58	4.90	26	3.39	0.0023

Best fit mixed-effect models. Frame by frame analysis. Mixed-effect model for the distance face-to-gaze level, in the facial expression condition, before, and after the onset of the social cue. The analysis is performed frame by frame, (a frame corresponds to 40 ms). Fixed effects: frameID, temporal distance from the onset of the facial expression, expressed in frame number; AUT, autism. The model provided by AUT is the best explanation for the differences in gaze-to-face distances present in the data, after the onset.

After the onset of the facial expression, only autism (AUT) has a significant effect on the measured gaze-to-face distances, see Table 4. This means that the difference in temporal gaze behaviors is mostly explained by the autism rather than by the amount of time that passed from the onset of the facial expression. Specifically, the two groups significantly differ (*p* = 0.0018) in the gaze-to-face distance after the onset. Considering the whole segment, there is no effect (*p* > 0.5) of the frameID and no interaction effect (*p* > 0.5) between autism and frameID (AUT^*^frameID). Therefore, the amount of time passed from the onset of the facial expression, does not explain the difference in the measures of the distances, when considering the whole segment, after the onset.

The statistics for the best resulting model, i.e., the one explained by the AUT only, are reported in Table 5. This shows that, after the onset of facial expression, the gaze-to-face distance varies significantly according to the autism factor (AUT). More specifically autistic children attend to the adult's face less (*M* = 38.58° of eccentricity, *SD* = 3.37°) than the TD children (*M* = 22.00° of eccentricity, *SD* = 4.90°).

**Figure 5:**
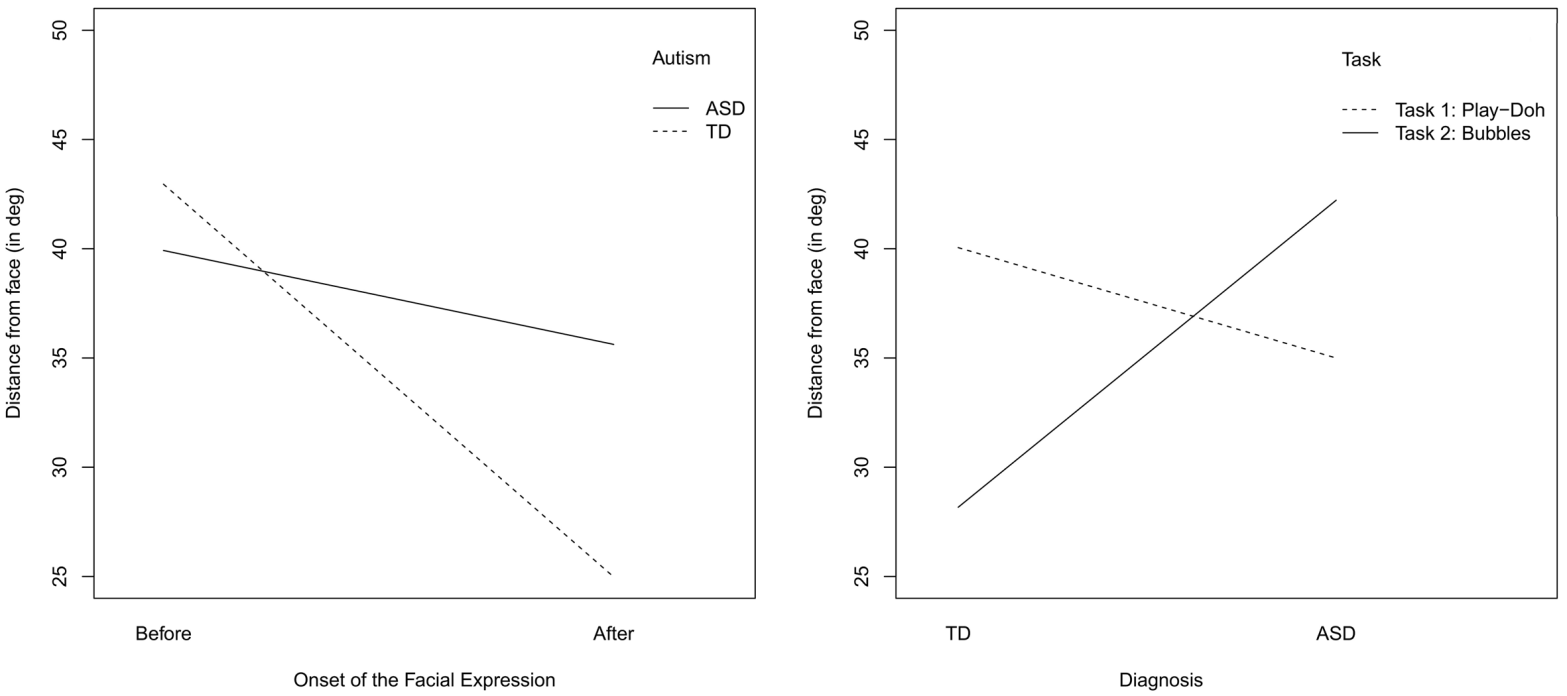
**Facial expression condition: interaction plots of the mean value of the gaze-to-face distance, computed over temporal intervals before and after the onset of the facial expression**. Both the intervals before and after the onset correspond to temporal window of 1200 ms (i.e., 30 frames).

To further visualize the attention shifts just after the onset of the facial expression, we represent the mean value of the gaze-to-face distance computed over the whole duration of the episode prior and following the onset of the social cue, for the two groups of children, see Figure 5, on the left. The interaction plots confirm the results that we have already assessed by fitting the linear mixed-effect models in Tables 3–5. Specifically, both the children with ASC and the TD children look at the adult's face from a mean distance that is greater before than after the facial expression onset. Moreover, only after the facial expression has occurred, does the mean gaze-to-face distance differ in the two groups; the gaze is closer to the AOI of the adult's face for the TD children than for the children with ASC. Thus, both children orient to the social visual stimuli though the children with ASC do that to a lesser extent. In fact, in Table 3, we reported a significant interaction effect (*p* < 0.0001) between onset and autism (ONS^*^AUT).

Figure 5 displays also the interaction effect between task and autism. Despite the fact that, in the video fragments included in the analysis, the objects of interaction are not present in the field of view, we can observe from Figure 5 (right) that they affect the pattern of visual attention. During the bubbles task (TASK 2), the TD children tend to look at the adult's face more than when they play the Play-Doh game (TASK 1). This trend can be simply explained by the nature of the tasks. Specifically, the bubbles task (TASK 2) requires the adult to blow off bubbles. As children watch the bubbles float away from the adult's mouth, they keep their gaze close to the adult's face. Interestingly, children with ASC show the opposite pattern: they look at faces more than TD children while they are playing Play-Doh and look at faces much less than TD when they are playing with the bubbles. Thus, they seem to avoid the faces when there is more opportunity to attend them. Again, the patterns shown in Figure 5 are confirmed by the fit provided in Table 3. There is reported a significant (*p* < 0.0001) double interaction effect between onset and autism and between autism and task (ONS^*^AUT^*^TASK).

### 3.2. Analysis of first fixation after the facial expression onset

No significant main effects are found for any of the factor. For the sake of conciseness we report only the linear model fitted for the autism factor (AUT).

Both the TD children (RTs: *M* = 329.69, *SD* = 51.57 ms) and children with ASC (RTs: *M* = 361.45, *SD* = 71.82 ms) show similar reaction times (*df* = 197; *p* = 0.6619, AIC = 1396.946; BIC = 1410.08; logLik = −694.47; Residuals = 7.67; Correlation = −0.72) when presented with facial expressions and in absence of any distractors.

In addition, the TD children (FFLs: *M* = 561.83, *SD* = 37.09 ms) and children with ASC (FFLs: *M* = 511.11, *SD* = 55.15 ms) spend similar amount of time when fixating the adult's face for the first time after the facial expression's onset (*df* = 197; *p* = 0.3662, AIC = 1470.69; BIC = 1483.82; logLik = −731.34; Residuals = 9.68; Correlation = −0.67), in absence of any distractors.

### 3.3. Spatio-temporal characteristics of gaze patterns in response to speech

**Figure 6:**
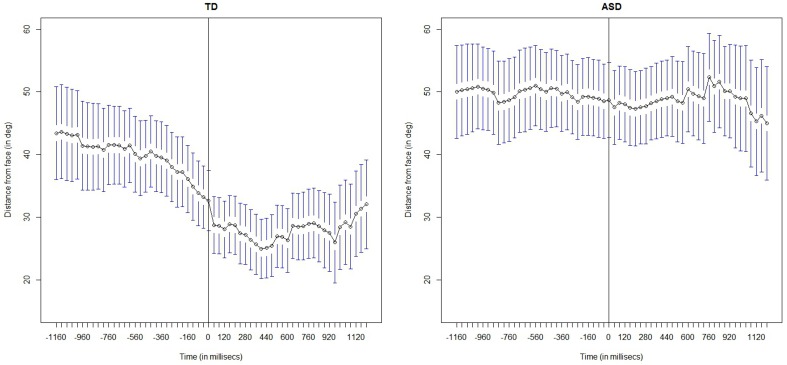
**Speaking condition: frame-by-frame plot of the face-to-gaze distance, for the TD children (on the Left) and for the children with ASC (on the Right)**. Mean and quartiles of the temporal profiles the viewing patterns are depicted for each frame, 1200 s (30 frames) before and after the adult's onset of the speech. The vertical line in the middle highlight the onset of the social cue.

Figure 6 displays the evolution of the gaze-to-face distance, before and after the onset of the speech stimulus, in the speaking condition. The pattern of visual behavior in TD children is similar to that observed in the facial expression condition: TD children start looking at the adult's faces more after than before the onset of the speech. We can observe that they shift their gaze rapidly to the face immediately after the onset of the speech event. ASC children differ drastically in their visual pattern. They seem to not attend to the adult's face following the speech event; unaffected by the social event.

To assess quantitatively the dependency of the evolution of the gaze-to-face distance measure on the onset of the speech (ONS), on task (TASK), autism (AUT) and chronological age (AGE), we computed different linear mixed-affect models to account for the strength of the dependency on each of these factors, see Table 6. We recall that the onset factor (ONS) separates all gaze-to-face distances into two groups: before the onset and after the onset.

**Table 6:** **Speaking condition**.

	**Model**	***df***	**AIC**	**BIC**	**logLik**	**Test**	**L.Ratio**	***p*-value**
m1	Null Model	3	93366.04	93387.71	−46680.02			
m2	AUT	4	93354.20	93383.08	−46673.10	m1 vs. m2	13.85	<0.0001
m3	AUT + ONS	5	93201.92	93238.03	−46595.96	m2 vs. m3	154.27	<0.0001
m4	AUT ^*^ ONS	6	93083.76	93127.09	−46535.88	m3 vs. m4	120.15	<0.0001
m5	AUT ^*^ ONS + TASK	8	93022.82	93080.59	−46503.41	m4 vs. m5	108.35	<0.0001
m6	AUT ^*^ ONS ^*^ TASK	14	92862.71	92963.81	−46417.35	m5 vs. m6	172.11	<0.0001

Best fit mixed-effect models. Average distance before and after the onset. Mixed-effect models providing the best fit for explaining the face-to-gaze distance levels, in the speaking condition.

The analysis is performed by taking into account the average distance before and after the onset of the speech.

Fixed effects: AUT, autism; ONS, onset of the facial expression; TASK, game (Play-Doh or bubbles).

We find that the autism factor (AUT) best increases the quality of the fit compared to the null model. BIC, AIC (i.e., the lowest, the best) and log likelihood (i.e., the highest, the best) criteria indicate a strong preference for the model including autism relative to the initial model. Note that there is a significant difference (*p* < 0.0001) in the gaze-to-face distances between children with ASC and TD children. The diagnosis provides the best explanation in the differences presented by the measures of the gaze-to-face distances. In fact, it has been selected as the model that best describes the differences in the dependent variable. We recall here that for the sake of conciseness we only report best fits in tables.

The onset factor (ONS) and the task factor (TASK) further increase the quality of the fit of the model (*p* < 0.0001, AIC decreases). However, more significant improvements (*p* < 0.0001, great AIC decrement) to the models are obtained by examining the interactions effects (AUT^*^ONS and ONS^*^TASK). This indicates that the gaze-to-face distance is affected by the onset condition and the type of task, in both children with ASC and TD children.

To quantitatively assess the differences between the two groups after the onset of the speech, we further compute several linear mixed-effect models considering the gaze-to-face distances frame by frame, after the onset separately. The analysis frame by frame considers the set of measures on the face-to-gaze distance in each specific frame, i.e., for each interval of 40 ms (the sampling period of the cameras).

We report in Table 7 on the comparison between three different models in order to show that distance face-to-gaze is related only to the diagnosis and not to the amount of time passed from the onset of the speech, i.e., the starting of the speaking event. In fact, adding autism (AUT) to the null model significantly improves the fit (*p* = 0.0002, AIC decreases).

**Table 7:** **Speaking condition**.

**After the onset**								
	**Model**	***df***	**AIC**	**BIC**	**logLik**	**Test**	**L.Ratio**	***p*-value**
m1	Null Model	3	45776.16	45795.73	−22885.08			
m2	AUT	4	45763.95	45790.05	−22877.97	1 vs. 2	14.21	0.0002
m3	AUT + frameID	5	45763.58	45796.21	−22876.79	2 vs. 3	2.36	0.1241
m4	AUT ^*^ frameID	6	45764.60	45803.75	−22876.30	3 vs. 4	0.98	0.3211

Mixed-effect models. Frame by frame analysis. Mixed-effect models for the distance face-to-gaze level, in the speaking condition, after the onset of the social cue. The analysis is performed frame by frame, (a frame corresponds to 40 ms). Fixed effects: frameID, temporal distance from the onset of the speech, expressed in frame number; AUT, autism.

The model in Table 8 also measures the relationship between the autism factor (AUT) and the gaze-to-face distance. The results reported in Table 8 show that, for the TD children, the mean gaze-to-face distance is 22.31°; whereas for children with ASC the mean gaze-to-face distance is 46.57°. Therefore, there is a difference of 24.26°, (*SD* = 5.69), between the two groups. Specifically, the ASC children seem to avoid adult's face: they look at the adult's face significantly less than TD children and the difference in the mean gaze-to-face distance is great.

**Table 8:** **Speaking condition**.

**After the Onset, ASC, and TD, Best fit Model**					
	**Value**	**Std.Error**	***df***	***t*-value**	***p*-value**
(Intercept)	22.31	3.77	5013	5.90	<0.0001
AUT	24.26	5.69	28	4.26	<0.0001

Mixed-effect models. Frame by frame analysis. Mixed-effect models for distance face-gaze level, in the speaking condition, after the onset of the social cue. The analysis is performed frame by frame, (a frame corresponds to 40 ms); AUT, autism. Fixed effects: frameID, temporal distance from the onset of the speech, expressed in frame number.

**Figure 7:**
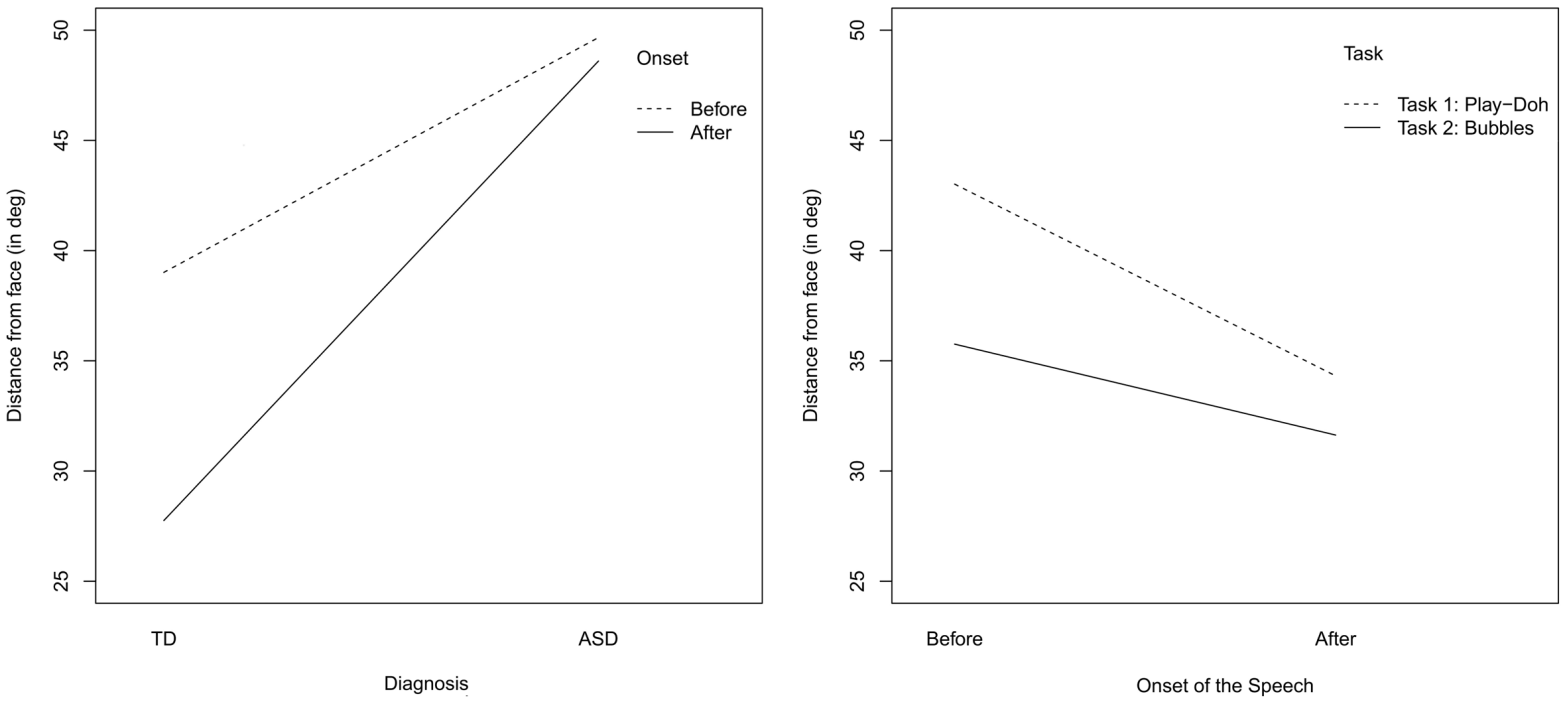
**Speaking condition: interaction plots of the average value of the gaze-to-face distance, computed over temporal intervals before and after the onset of the speech**. Both the intervals before and after the onset correspond to temporal window of 1200 ms (i.e., 30 frames).

To visually observe the interaction effects between the autism and onset factors (AUT^*^ONS), we report the corresponding interaction plot in Figure 7. We observe that the mean gaze-to-face distance is shorter for the TD children during the period following the onset of the speech, i.e., when the adult is speaking (after onset) than prior to the beginning of the speech (before onset). This confirms the result obtained with the model fitting: it indicates that TD children look at faces more after than before the onset. Children with ASC show the opposite trend: they seem to not attend to the adult's face after the speech (after onset). In addition, children with ASC look at faces less than TD children, after the onset of the speech stimulus. Again, the plots replicate the results obtained with by fitting the mixed-effect models.

Figure 7 shows the interaction between the onset and the tasks (ONS^*^TASK). For both the tasks and the groups (ASC vs. TD), the mean distance of the gaze to the adult's face is smaller after the onset of speech than before the onset. However, for the task involving the use of the Play-Doh, (TASK 1), the distance is much greater than for the bubble task before the onset. These gaze behaviors again agree with the kind of task involved, as discussed previously. In the Play-Doh session, children usually look down at the table in front of them, watching either the object held by the adult or the piece of Play-Doh compound they are manipulating. Conversely, during turn taking sessions of bubble shooting, gaze more likely spans in the vicinity of the adult's face as shot bubbles start by appearing close to the face. The interaction plot is in agreement with the results in Table 7 about the double interaction between autism and onset and onset and task, (AUT^*^ONS^*^TASK).

### 3.4. Analysis of first fixation after the speech onset

We herein report the analysis of the collected set of RTs in the **speaking condition**. The best fit for the model is found for the autism factor (AUT). No other main or interaction effects are found. The children with ASC (RTs: , *M* = 531.7, *SD* = 59.42 ms) orient to speech stimuli slower (*df* = 192; *p* = 0.001, AIC = 1377.99; BIC = 1391.02; logLik = −684.9948) than TD children (RTs: *M* = 302.33, *SD* = 39.17 ms), in the absence of any distractors. However, the TD children (FFLs: *M* = 561.83, *SD* = 50.74 ms) and the children with ASC (FFLs: *M* = 467.63, *SD* = 75.67 ms) spend similar time in fixating for the first time the adult's face after the speech onset, (*df* = 192; *p* = 0.2239, AIC = 1410.23; BIC = 1423.266; logLik = −701.11182; Residuals = 8.75; Correlation = −0.67), in the absence of any distractors.

## 4. Discussion

Our results reveal significant differences between children with ASC and TD children. Both groups of children show overt shifts of visual attention toward the adult's face in response to facial expressions, specifically smiles and facial expressions of surprise. However, the children with ASC differ from the TD children in that, even though they look at the adult's face, they do so to a lesser extent. In particular, this tendency is evident during the bubbles task in which there would be more opportunity to look at faces, since bubbles float away from the adult's mouth.

Impaired attention to facial expressions is confirmed by several studies conducted with eye-tracking technology that demonstrated, for instance that children with ASC look at the eyes in faces presenting facial expressions less often than TD children (Pelphrey et al., 2002; Corden et al., 2008).

To the best of our knowledge, Sasson et al. (2007) conducted the only study that focuses on social orienting to facial expressions. They compare the gaze behaviors of adults with autism, with those of adults with schizophrenia and typically developed peers. The subjects observe a series of complex social scenes where facial expressions of emotion are either included or digitally erased [an experimental paradigm designed by Adolphs and Tranel (2003)]. They find out that the subjects both with autism and with schizophrenia look directly at faces less than the TD individuals. In addition, they find that only subjects with autism orient at the same velocity when emotionally meaningful informations are both presented and erased. However, the authors do not find any difference in the latency to look at the adult's facial expressions between the TD group and the subjects with autism.

We replicated the results in overt orienting to facial expressions reported by Sasson et al. (2007) in dynamic and complex situations where children are playing and interacting with the adults. Our results show that when presented with real-life interactions, children with autism are spontaneously attracted to facial expressions, even though to a lesser extent than TD children. In fact, no child was instructed to look at the adult's face prior to the experimental sessions. Thus, we presented an experimental paradigm in which the naturalness of a subjects' response is not reduced by the fact that the experimenter selected the target of the attention for the subject, an issue raised by Birmingham et al. (2012).

Similarly, to Sasson et al. (2007) we find that the latency and the duration of the first fixation to the facial expressions are the same in the two groups of children. In our experimental paradigm, neutral faces precede the onset of the facial expressions, so the children had the opportunity to either overtly or covertly attend to them before the onset of a facial expression. However, we computed the reaction times by taking into account only segments in which the children did not look at the adult's face before the onset. Therefore, we hypothesize that children could orient to a (silent) facial expression only because they saw it while they covertly attending to the facial expression's onset.

This hypothesis is in agreement with several studies reporting intact abilities in visual covert orienting (Chawarska et al., 2003; Swettenham et al., 2003; Iarocci and Burack, 2004; Senju et al., 2004; Ristic et al., 2005; Vlamings et al., 2005) and in peripheral vision tasks (Yoshida et al., 2011) of individuals with autism.

Given the lesser extent to which children overtly orient to faces we suggest also that individuals with ASC may be impaired in using informations from faces and present particular strategies for attending to these social cues. This last hypothesis was also raised by other authors (LeCouteur et al., 1989; Lord et al., 1994; Filipek et al., 1999; Lord et al., 2000; Sasson et al., 2007; Harms et al., 2010).

In this study, we are not able to assess the accuracy in recognizing emotion when facial expressions are presented, because we did not ask children to judge the facial expressions they were exposed to. Sasson et al. (2007) observe that, whereas adults with schizophrenia present slower orienting to faces than the other groups, they recognize the presence of faces faster than the individuals with ASC.

Research that investigated the ability to recognize emotions from facial expressions in autism, reported mixed findings Harms et al. (2010). Some studies highlight the impairments in facial expression recognition tasks among participants with ASC (Hobson, 1986; Bormann-Kischkel et al., 1995; Buitelaar et al., 1999; Celani et al., 1999; Gross, 2004; Grossman and Tager-Flusberg, 2008). Whereas, others revealed similar facial recognition skills in TD and ASC groups (Prior et al., 1990; Capps et al., 1992; Grossman et al., 2000; Adolphs et al., 2001; Gepner et al., 2001; Robel et al., 2004; Castelli, 2005; Rosset et al., 2008; Jones et al., 2010). Other studies find impairments in the ability of ASC people in recognizing emotions with negative valence such as fear and anger (Howard et al., 2000; Ashwin et al., 2006).

Our results show that when the adult speaks, the children with ASC look at the adult's face less than TD children. However, in presence of speech we observe that children with ASC avoid faces more than when the adult makes facial expressions. In fact, the best fit for the the temporal gaze-to-face distances is accounted for by the diagnosis and not by the onset of the speech (see Table 6).

This result confirms the observation reported in the literature on autism and assessed through the analysis of video recordings, i.e., impairments in social orienting to speech in real life situations (Dawson et al., 1998; Werner et al., 2000; Dawson et al., 2004).

Moreover, we observed that when children with ASC orient to the adult's face after the speech onset, they are slower than TD children, though their first fixations to the adult's face last a similar amount of time than that observed in TD children.

These findings may be attributed to deficits in speech auditory processing. In fact, there is evidence that individuals with ASC show severe deficits in their ability to process social cues when communicating and interacting with others (Rapin and Dunn, 2003; Siegel and Blades, 2003), especially when physically complex social stimuli are involved. For instance, the inability to elaborate social sounds, but not tones, is assessed in children with ASC (Ĉeponienė et al., 2003; Kuhl et al., 2005; Oram-Cardy et al., 2005; Whitehouse and Bishop, 2008). Klin conducted (Klin, 1991; Klin et al., 1992) tests of listening preferences in children with autism, showing that typical and developmentally delayed children select their mother's voice over the noise of many superimposed voices, whereas children with ASC present the opposite behavior (Klin, 1991; Klin et al., 1992). Deficits in processing speech prosodies (Kujala et al., 2005; see Peppe and McCann, 2003, for a review) were reported in both children and adults with autism. Evidence of abnormal perception and processing of speech sounds in the ASC population is reported by functional imaging studies (Boddaert et al., 2004; Bomba and Pang, 2004). Atypical and inadequate responses to social sounds in autism, are likely to arise from an abnormal auditory cortical processing, as suggested by Boddaert et al. (2004). In their study, they find low level of activation in the left hemisphere in response to speech stimuli, and abnormally dominant processing in the right hemisphere. The reverse pattern was found in the comparison group. Moreover, in our settings the difficulty in speech processing may be exacerbated by the presence of social visual informations (e.g., facial expressions and mouth movements) and thus also due to the difficulties in the processing of multi-modal sensorial information found in autism (see Iarocci and McDonald, 2006, for a review).

Our study complements the literature dedicated to attention orienting by providing information on how ASC people attend to social stimuli in naturalistic interactions with familiar adults. Thanks to the broad field of view recorded by the WearCam eye-tracker and to its portability and unobtrusiveness, we were able to focus on what was present in the child's field of view in an unconstrained, naturalistic environment.

Our study is limited in the number and type of facial expressions displayed by the adult and do not distinguish across these facial expressions. It is also limited in the number of subjects and did not pursue a detailed analysis of the content of the speech uttered by the adult. This study hence provides only a set of observations that will need to be assessed through more extensive exploration with larger groups of subjects in the future. However, this study has value in that it offers a unconstrained paradigm to the study of visual orienting patterns in children with ASC, in naturalistic live dyadic interactions. To our knowledge, this is the first comparative assessment of attention shifts to faces in the presence of two distinct social stimuli in naturalistic settings. Thanks to the use of this novel wearable eye-tracker, we provide a quantitative assessment of the temporal evolution of the gaze in the broad field of view.

## 5. Conclusion

The main aim of our study is to investigate whether children with Autism Spectrum Conditions (ASC) spontaneously orient to people's face during dyadic playing interactions with adults, in naturalistic settings. In order to identify patterns of orienting to faces, we conducted an analysis of the visual temporal patterns displayed by children with ASC and typically developing (TD) children, in response to social stimuli. We sought to examine whether the complexity of the social stimuli and the demands of the task, play a role in the behavioral patterns of children with ASC in attention orienting to social cues. In our experimental protocol we mainly focus on the spontaneity of the interactions between the adults and the children. During the play sessions (respectively, with the Play-Doh and bubbles), the adult's were only asked to take turns in playing with the children.

For testing the tendency to orient to social stimuli in children, we designed a behavioral coding system based on similar studies that analyzed the attention behaviors in naturalistic interactions (Osterling and Dawson, 1994; Dawson et al., 1998; Baranek, 1999; Werner et al., 2000; Wimpory et al., 2000; Osterling et al., 2002; Dawson et al., 2004; Werner and Dawson, 2005).

Similarly to the studies conducted on retrospective analysis of home videotapes, we used a cue to highlight the presence of a specific event during the interaction. We examined the gaze patterns with respect to the target, i.e., the visual or visual-auditory social stimuli target, that occurs after the cue: the onset of the social stimuli. Differently from experimental paradigms conducted in laboratory settings, we did not ask the subjects to give their attention somewhere after the presentation of the cue and before the target; nor did we ask the subjects to specifically give attention to the target. Our aim is in fact to register and quantify the presence of spontaneous orienting to social cues in unconstrained, natural environments.

We separated the video segments containing two main events: facial expressions (expressing either surprise or joy) and speech utterances made by the adult's. Each fragment that is taken into account in the statistical analysis had to respect the following pattern: a segment in which the adult is silent and shows a neutral facial expression, precedes a segment in which the adult is either speaking or making facial expressions. No video fragments were taken into account in the analysis which contained objects in the child's field of view. The rationale here is to avoid any biases in the children's looking strategies. The presence of a neutral facial expression before the onset of the social stimuli enables us to quantify the change in the visual behavior that takes place after the onset of the social cue.

In this study we analyze shifting of attention to faces in children with ASC during playful dyadic social interaction. We contrasted a group of children with ASC from 2 to 11-years old and a group of TD children between 3 and 6-years old. We compared the two groups with respect to their temporal gaze behaviors in two kinds of social interaction: an adult's face that speaks and an adult's face showing facial expressions of emotion when no distractors were in the child's field of view. Similarly to TD children, children with ASC shift their gaze toward the adult when the adult made a facial expression; though their tendency to shift the attention to the social cue is less marked than in the other group. In contrast to TD children, children with ASC shift their gaze toward the adult when the adult was talking significantly less. The impairment in social orienting to speech sound seems more severe than that observed with facial expressions.

Children with ASC orient to speech slower than TD children. This behavior is probably due to the difficulties in elaborating social sounds (Ĉeponienė et al., 2003; Kuhl et al., 2005; Oram-Cardy et al., 2005; Whitehouse and Bishop, 2008) and in processing multi-modal sensorial information (see Iarocci and McDonald, 2006, for a review). In fact, in our experiments the social auditory stimuli (i.e., sounds of the speech) is co-present with the visual stimuli (i.e., the facial expressions involved in the speech).

Additionally, children with ASC show intact ability in visual covert shifting of attention when they look at faces containing facial expressions. This confirms the evidence of an intact covert attention in individuals with autism reported in the study conducted by Sasson et al. (2007) on orienting to facial expressions and by the results assessed through visual cueing tasks (Chawarska et al., 2003; Iarocci and Burack, 2004; Kylliäinen and Hietanen, 2004; Greenaway and Plaisted, 2005; Ristic et al., 2005; Bird et al., 2006).

Even though the results are encouraging and of importance, we believe this represents only a preliminary study and suggest further investigations that test whether similar results in attention orienting may be replicated in a greater group of children with ASC.

### Conflict of interest statement

The authors declare that the research was conducted in the absence of any commercial or financial relationships that could be construed as a potential conflict of interest.
